# Universal Logic-in-Memory Gates Using Reconfigurable Silicon Transistors

**DOI:** 10.3390/mi16121348

**Published:** 2025-11-28

**Authors:** Sunhyuk Kim, Nahyeon Kim, Yaeyeon Ko, Doohyeok Lim

**Affiliations:** School of Electronic Engineering, Kyonggi University, Suwon 16227, Republic of Korea

**Keywords:** polarity gate, control gate, universal logic-in-memory gate

## Abstract

This study aims to implement universal logic gates using polarity control within a single silicon transistor structure. For this purpose, a reconfigurable transistor based on a p-i-n structure featuring two polarity gates (PGs) and one control gate was proposed, and its electrical characteristics and logic-in-memory (LIM) circuit operations were analyzed via two-dimensional technology computer-aided design simulations. The proposed device could be perfectly reconfigured into p-channel or n-channel modes because virtual doping effects could be induced according to the polarity of the PG voltage. Moreover, based on the positive feedback and latch-up phenomena, a steep subthreshold swing of approximately 1 mV/dec and a high ON/OFF current ratio of the order of 10^10^ were achieved. Building on these characteristics, we successfully verified NAND LIM operation in the p-channel mode and NOR LIM operation in the n-channel mode by connecting two of the proposed devices in parallel. The reconfigurable silicon transistor proposed in this study could perform both NAND and NOR LIM operations while sharing the same device structure and can be expected to play a key role in implementing high-density, low-power LIM systems in the future.

## 1. Introduction

**Figure 1:**
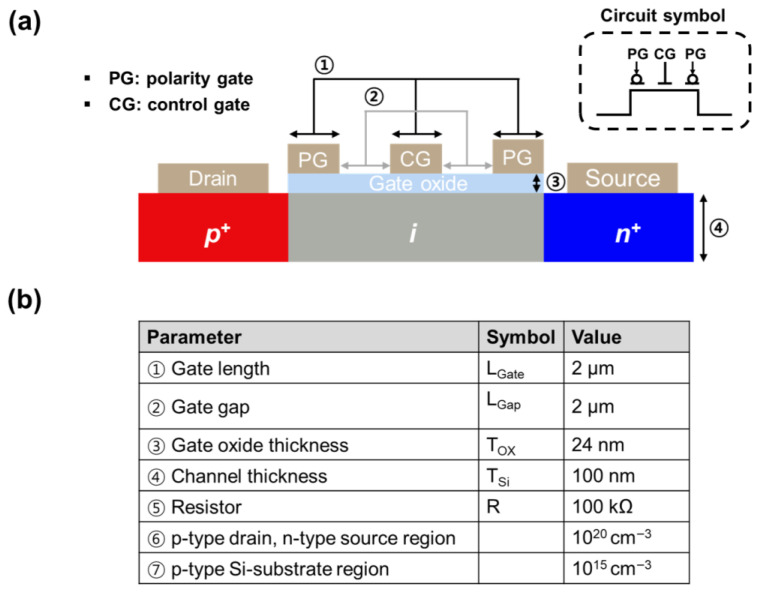
(**a**) Schematic cross-section and circuit symbols of the proposed reconfigurable silicon transistor. (**b**) Key structural and electrical parameters used in the TCAD simulation, including the gate length, oxide thickness, and doping concentrations.

In modern computing, where data-intensive applications such as artificial intelligence and big data analytics are becoming increasingly important, the von Neumann architecture suffers from latency and high power consumption owing to frequent data transfers, resulting in a performance bottleneck [1]. This problem arises from the physical separation of the processor and memory inherent in the von Neumann architecture. Consequently, logic-in-memory (LIM) architectures have been explored [2,3,4]. LIM architecture enable both logic and memory operations within a single physical block, eliminating the need for frequent data movement and thereby improving computational speed and energy efficiency. This approach has garnered considerable attention as a promising strategy for next-generation, low-power, high-efficiency computing applications. Although various devices, such as resistive random-access memory and magnetic random-access memory, have been proposed for implementing LIM architecture, they suffer from high operating voltages and complex control mechanisms. Additionally, static random-access memory (SRAM)-based LIM structures offer the advantage of high-speed operation but are limited in terms of their integration density and leakage current [5,6]. Recently, feedback field-effect transistors (FBFETs) based on a positive-feedback mechanism have garnered attention as promising devices for LIM applications [7,8,9]. The positive-feedback mechanism controls the charge carrier injection according to the potential barrier, operating the device through the formation of a positive-feedback loop [10]. This mechanism enables FBFETs to achieve abrupt switching at low operating voltages while exploiting hysteresis characteristics for simultaneous memory operation [11,12]. Moreover, as shown in Figure 1a, the reconfigurable nature of the device—achieved by controlling the polarity of the three gates—enables channel selection according to the majority carrier, making it suitable for LIM applications [13].

In this study, we implemented NAND and NOR gates capable of simultaneous logic and memory operations using a three-gate transistor. The gates located at both ends of the transistor function as polarity gates (PGs) that determine the channel mode according to the polarity of the applied voltage. The centrally located gate serves as a control gate (CG), regulating the injection of charge carriers. The operation of the device is determined by the applied voltage. In p-channel mode, a positive voltage is applied to the drain, whereas in n-channel mode, a negative voltage is applied to the source. Using this operating scheme and configuring the transistor with a resistor in a pull-up and pull-down structure, logic functions, such as NAND and NOR, and memory operations can be simultaneously implemented within a single device.

## 2. Materials and Methods

The reconfigurable silicon transistor used in this study was based on a p-i-n structure, as illustrated in Figure 1a. The three gates located in the intrinsic (i) region consist of two side polarity gates and one central control gate. The two polarity gates are electrically coupled. It was fabricated on a p-type silicon substrate (doping concentration of 10^15^ cm^−3^) and featured a heavily doped p^+^ drain (10^20^ cm^−3^), a heavily doped n^+^ source (10^20^ cm^−3^), and an intrinsic silicon channel of thickness (*T_Si_*) 100 nm. Two PGs and one CG were positioned on the gate oxide above the intrinsic channel. The gate oxide thickness (*T_ox_*) was 24 nm. Both the gate length (*L_G_*) for all gates and the gap between gates were 2 μm. Two-dimensional (2D) device simulations were performed using the Silvaco technology computer-aided design (TCAD) toolset to analyze the device characteristics. The device structure was generated using Silvaco Athena, and the electrical characteristics were simulated using Silvaco Atlas in mixed mode. The following physical models were employed in the simulations to ensure accuracy—that is, the Fermi–Dirac statistics [14] (Fermi), Lombardi mobility [15], parallel electric field dependence for high-field carrier velocity [16], bandgap narrowing (BGN) [17], concentration-dependent Shockley–Read–Hall recombination, Auger recombination (Auger) [18], and Klaassen mobility [19] models.

## 3. Results and Discussion

**Figure 2:**
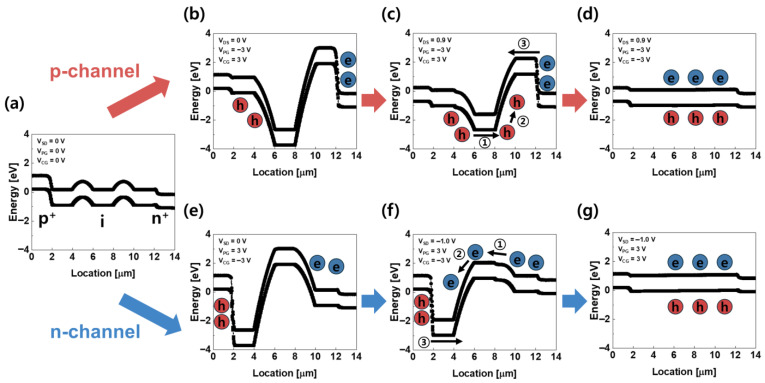
The energy band diagrams for the p-channel (**b**–**d**) and n-channel (**e**–**g**) operation modes, depending on the majority carriers. (**a**) illustrates the initial energy band structure with no voltage applied to any terminal, presenting the basic p⁺–i–n⁺ configuration and the corresponding initial potential profile. In the p-channel mode, (**b**) represents the initial potential barrier that suppresses hole injection from the drain side, (**c**) shows an intermediate state where the barrier at the drain is partially lowered by the applied gate bias, and (**d**) presents the feedback-on state in which accumulated holes further reduce the tunneling barrier at the drain, triggering rapid switching through positive feedback. In the n-channel mode, (**e**) shows the initial barrier that blocks electron injection from the drain side, (**f**) depicts an intermediate state where the drain-side barrier becomes partially relaxed under bias, and (**g**) illustrates the feedback-on state where accumulated electrons lower the drain-side barrier further, forming a positive-feedback loop that results in abrupt turn-on behavior..

Figure 2 shows the device energy band diagrams for both p-channel and n-channel operations, depending on the majority carriers. The transistor proposed in this study controls the on/off behavior of the device by adjusting the potential barrier height of the charge through three gate voltages. In the OFF state, a potential barrier is formed for both electrons and holes, which inhibits the flow of current. On the other hand, in the ON state, this barrier is lowered, allowing electrons and holes to be injected into the channel region, and the interaction between them creates a positive feedback loop. Figure 2a shows the energy band diagram of the initial state without any applied bias, exhibiting a p^+^–i–n^+^ doping profile. When *V_PG_* = −3 V and *V_CG_* = 3 V are applied in the initial state, the energy bands beneath each gate become virtually p^+^–p–i–p–n^+^, as shown in Figure 2b, forming potential barriers for both holes and electrons and thereby preventing their injection into the channel. When *V_DS_* = 0.9 V, the energy band in the drain region is lowered, reducing the potential barrier for holes. However, owing to the potential barrier, holes in the drain aren’t injected into the channel, as shown in Figure 2c. As *V_CG_* is swept from 3 V to −3 V, the hole potential barrier is reduced, allowing holes to be injected into the channel. The increased accumulation of holes in the potential well subsequently lowers the electron potential barrier, enabling electron injection. As a result, the potential barriers for both carriers are reduced, as shown in Figure 2d, forming a positive feedback loop that allows current flow. This band modulation demonstrates the p-channel operation in which holes serve as the majority carriers.

Conversely, when a positive voltage of 3 V is applied to *V*_PG_ and a negative voltage of −3 V is applied to *V*_CG_ in the initial state of Figure 2a, the device becomes virtually p^+^-n-i-n-n^+^ doped, as shown in Figure 2e, forming potential barriers for both holes and electrons. In the n-channel operation, applying a negative voltage to *V_SD_* (*V_SD_* = −1.0 V) raises the energy band at the source, reducing the potential barrier for electrons. However, owing to the potential barrier, electrons in the source aren’t injected into the channel, as shown in Figure 2f. As the voltage applied to *V*_CG_ is swept from −3 V to 3 V, the potential barrier for electrons decreases. As a result, electrons are injected into the channel and accumulated in the potential well. This enhances electron injection, increasing the number of electrons accumulated in the potential well. Consequently, as shown in Figure 2f, the potential barrier for holes decreases, allowing holes to flow toward the source and electrons toward the drain. This carrier flow induces a positive feedback loop in which electrons act as the majority carriers, thereby implementing the p-channel operation mechanism and generating channel current.

**Figure 3:**
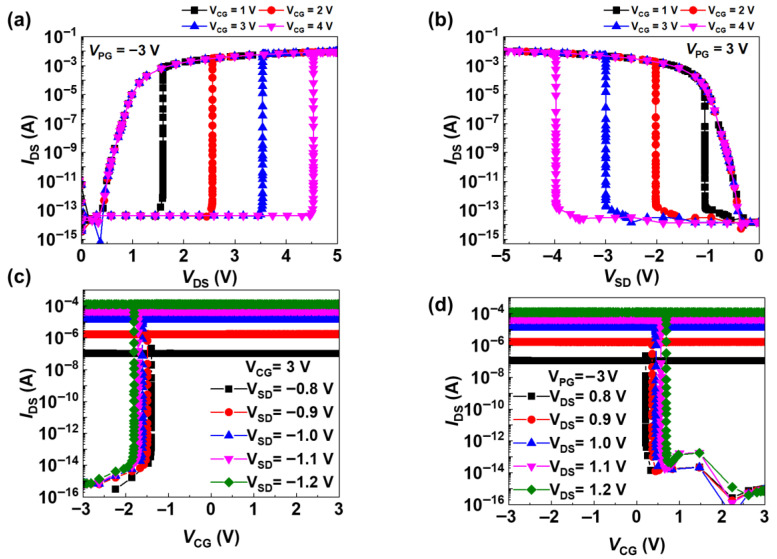
Electrical characteristics of the transistor: output characteristics for the (**a**) p-channel and (**b**) n-channel under different control gate voltages, and transfer characteristics for the (**c**) p-channel and (**d**) n-channel as a function of the drain–source voltage.

Figure 3a,b show the *I_DS_* characteristics of *V_DS_* in the n-channel and p-channel operations. Applying −3 V to *V_PG_* results in the device being virtually doped as p^+^–p*–i–p*–n^+^. The potential barrier formed by the applied voltage prevents the injection of holes and electrons into the channels. In this configuration, sweeping *V_DS_* from 0 to 5 V reduces the potential barrier for holes in the drain region. The injected holes accumulate in the potential well, lowering the potential barrier for electrons. This process initiates a positive-feedback loop, leading to an abrupt turn-on and latch-up.

As shown in Figure 3a, varying *V_CG_* from 1 to 4 V results in latch-up voltages of approximately 1.5, 2.5, 3.5, and 4.5 V, respectively. Figure 3b shows a device virtually doped with p^+^–n*–i–n*–n^+^ when 3 V is applied to *V_PG_*. Sweeping *V_SD_* from 0 to −5 V lowers the potential barrier for electrons in the source region, facilitating electron injection into the channel. These injected electrons accumulate in the potential well, thereby reducing the potential barrier for holes. Consequently, a positive-feedback loop is formed, leading to abrupt switching and latch-up behavior. The latch-up voltage increases with *V_CG_* and was observed to be approximately −1, −2, −3, and −4 V, respectively.

Figure 3c,d show the *I_DS_* characteristic according to *V_CG_* for the p-channel and n-channel operations of the transistor, respectively. The two graphs show that the steep switching of the device and clear separation of the ON and OFF states are bistable. Figure 3c shows the *I_DS_* transfer characteristics as a function of *V_CG_* in the p-channel mode with *V_PG_* fixed at −3 V. As *V_CG_* decreases from 3 to −3 V, the potential barrier in the drain region initially suppresses hole injection, but the barrier is lowered with increasing *V_CG_*, injecting holes into the channel region. Consequently, a positive-feedback loop is formed, leading to abrupt switching behavior with a subthreshold swing (SS) of approximately 1 mV/dec and an ON/OFF current ratio of the order of 10^11^.

Figure 3d illustrates that, in n-channel mode, the device turns on more rapidly as *V_DS_* increases. This behavior can be attributed to the enhanced electron injection, which facilitates internal potential changes and, in turn, promotes more rapid formation of the feedback loop. Under these conditions, the ON/OFF current ratio exceeds 10^11^, and the SS is 0.2 mV/dec.

**Figure 4:**
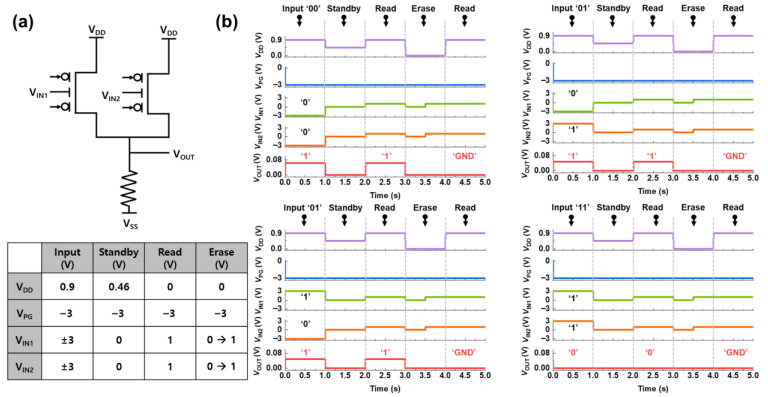
(**a**) Logic circuit diagram composed of two reconfigurable silicon transistors and a pull-down resistor. (**b**) AC simulation waveforms verifying the NAND LIM gate operation, showing the output signal *V_OUT_* in response to the input signals *V_IN1_* and *V_IN2_*.

Figure 4a shows the NAND circuit constructed using two reconfigurable silicon transistors in a parallel configuration and a pull-down resistor. A common voltage of −3 V was applied to the PGs of both transistors to set them to operate in p-channel mode. To receive external inputs, the CG of the left transistor is defined as *V_IN1_*, and the *V_CG_* of the right transistor is defined as *V_IN2_*. Voltage (*V_DD_*) is applied to the drain side of the parallel transistor. The operation of the circuit is determined by the positive-feedback mechanism within the transistors.

The AC simulation waveforms verified the NAND LIM operation, as shown in Figure 4b. When the voltage polarities of *V_PG_* and *V_CG_* are the same (e.g., *V_PG_* < 0 and *V_CG_* < 0), positive feedback is activated. If feedback is activated in at least one of the two transistors, the positive voltage *V_DD_* applied to the drain side is transferred to the output terminal *V_OUT_*, resulting in a positive output signal. This state is defined as logic “1.” Conversely, if the polarities of *V_PG_* and *V_CG_* are different for both transistors (e.g., *V_PG_* < 0 and *V_CG_* > 0), the feedback is deactivated. In this case, the *V_DD_* voltage is blocked, and the circuit is pulled to the ground state by the pull-down resistor, resulting in an output of 0 V at *V_OUT_*. This state is defined as logic “0.” The logical operation of the circuit corresponds to the truth table of the NAND gate. Based on the provided voltage conditions, a negative input (*V_IN_*) is defined as logic “0” (−3 V), and a positive input is defined as logic “1” (3 V). A positive output (*V_OUT_*) is defined as logic “1,” and an output of 0 V is defined as logic “0.” For input ‘00’ (*V_IN1_* = −3 V, *V_IN2_* = −3 V), the polarities of *V_PG_* (*V_PG_* < 0) and *V_CG_* (*V_CG_* < 0) are the same for both transistors, so the positive feedback is ON. Therefore, *V_DD_* is transferred to *V_OUT_*, resulting in a positive output (logic “1”). For input ‘01’ (*V_IN1_* = −3 V, *V_IN2_* = 3 V), the polarities of *V_PG_* (*V_PG_* < 0) and *V_CG_* (*V_CG_* < 0) are the same in the left transistor, so the feedback is ‘ON,’ and *V_OUT_* is positive (logic “1”). Similarly, for input ‘10’ (*V_IN_*_1_ = 3 V, *V_IN2_* = −3 V), the polarities of *V_PG_* (*V_PG_* < 0) and *V_CG_* (V_CG_ < 0) are the same in the right transistor, activating the feedback and resulting in a positive output (logic “1”). Finally, for input ‘11’ (*V_IN1_* = 3 V, *V_IN2_* = 3 V), the polarities of *V_PG_* (*V_PG_* < 0) and *V_CG_* (*V_CG_* > 0) are different for both transistors, so the feedback is ‘OFF.’ Consequently, *V_DD_* is blocked, and *V_OUT_* is 0 V (logic “0”). During the standby operation, *V_DD_* is lowered (*V_DD_* = 0.46 V) and each device retains its internal state. A device that has previously formed a positive-feedback loop preserves this condition, whereas a device that did not form the loop cannot regenerate a positive-feedback loop. As a result, the logic state defined during the input operation is maintained. During the read operation, *V_DD_* is increased to 0.9 V, and the previously stored logic state are retrieved as the positive feedback loop is reactivated. Conversely, is no device had formed a positive feedback loop during the input operation, the NAND logic circuit outputs the ground signal, consistent with the previous logic result.

For the erase operation, the *V*_DD_ is removed, and the control gate voltage *V*_CG_ is set to 0 V. Under these conditions, the accumulated carriers in the channel recombine, effectively eliminating positive feedback loops. As a result, during the following read operation, logic circuit outputs return to the ground level without any positive feedback loop.

**Figure 5:**
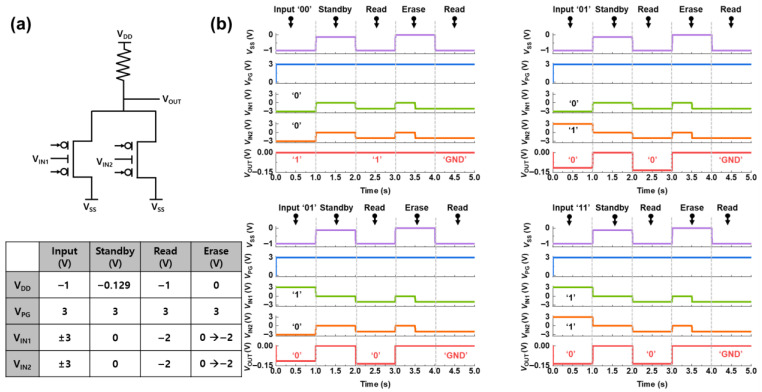
(**a**) Logic circuit diagram composed of two reconfigurable silicon transistors and a pull-up resistor. (**b**) AC simulation waveforms verifying the NOR LIM operation, showing the output signal *V_OUT_* in response to the input signals *V_IN1_* and *V_IN2_*.

The NOR circuit was constructed using two reconfigurable silicon transistors and one pull-up resistor, as shown in Figure 5a. A common voltage of 3 V was applied to the PGs of both transistors to set them to operate in n-channel mode. The *V_CG_* of the left transistor is defined as *V_IN1_*, and the *V_CG_* of the right transistor is defined as *V_IN2_* to receive external inputs. The operation of the circuit is determined by the positive-feedback mechanism within the transistors.

Figure 5b shows the AC simulation results used to verify the logical operation of the circuit. A negative input (*V_IN_*) is defined as logic “0,” and a positive input is defined as logic “1.” A negative output (*V_OUT_*) is defined as logic “0,” and an output of 0 V is defined as logic “1.” The simulation results were consistent with the NOR gate truth table. For input ‘00’ (*V_IN1_* = −3 V, *V_IN2_* = −3 V), the polarities of *V_PG_* (*V_PG_* > 0) and *V_CG_* (*V_CG_* < 0) are different for both transistors, so the positive feedback is ‘OFF,’ resulting in *V_OUT_* being 0 V (logic “1”). For input 01 (*V_IN1_* = −3 V, *V_IN2_* = 3 V), the polarities of *V_PG_* (*V_PG_* > 0) and *V_CG_* (*V_CG_* > 0) are the same in the right transistor, so the feedback is ‘ON’, and *V_OUT_* is approximately −0.15 V (logic “0”). Similarly, for input 10 (*V_IN1_* = 3 V, *V_IN2_* = −3 V), the polarities of *V_PG_* (*V_PG_* > 0) and *V_CG_* (*V_CG_* > 0) are the same in the left transistor, activating feedback and resulting in *V_OUT_* being approximately -0.15 V (logic “0”). Finally, for input ’11’ (*V_IN1_* = 3 V, *V_IN2_* = 3 V), the polarities of *V_PG_* (*V_PG_* > 0) and *V_CG_* (*V_CG_* > 0) are the same for both transistors, so the feedback is ‘ON,’ and *V_OUT_* is approximately −0.15 V (logic “0”). Subsequent the standby, read, and erase operation of the NOR gate circuit function based on the same principle as those of the NAND gate circuit. In the standby operation, *V_SS_* is set to −0.129 V, and the devices retain the previous logic results. In the read operation, *V_SS_* is decreased to −1 V, and the NOR gate circuits retrieve the previously stored logic states. In the erase operation, *V_SS_* and *V_CG_* are set to 0 V, and all formed positive feedback loops are removed. During the subsequent read operation, owing to the absence of the positive feedback loop, the NOR gate circuit outputs the ground signal.

The inconsistency stems from the fundamental difference in circuit. The NAND gate operates based on two pull-up network transistors where the bias is applied to the drain, whereas the NOR gate utilizes two pull-down network transistors where the bias is applied to the source. Consequently, these distinct schemes result in different output voltage levels for defining logic states. The proposed device has clearly defined On/Off states and steep switching characteristics, with bi-stable I–V behavior, which are tolerant to noise margin. In order to match the output voltage levels, applying a positive voltage to the drain enables the voltage levels of the NAND and NOR operations to be matched. Nevertheless, we intentionally applied a source bias instead of *V*_DD_. This operation enabled a significantly low operating voltage of 0.9 V, consistent with our goal of achieving low-power operation. In practical circuit-level operation, various device and process variations must be taken into account, which necessitates the use of a larger operating voltage. To secure a sufficient output voltage level under these conditions, circuit techniques such as employing larger load resistors or adding buffer stages are required. However, the primary objective of this demonstration was to showcase the potential for extreme low-power operation. To achieve this, we intentionally designed both the NAND and NOR circuits to operate at a very low supply voltage of 0.9 V, −1 V.

The difference in output voltage levels between the NAND and NOR gates arises from their distinct circuit configurations. In the NAND gate, two pull-up network transistors operate with a *V_DD_*, while the NOR gate employs two pull-down network transistors with a source-applied bias. These differing schemes lead to variations in the voltage levels that define logic states. The proposed device exhibits clearly defined on/off states and steep switching characteristics, along with bi-stable I–V behavior, providing robustness against noise margin issues.

To match the output voltage levels of the NAND and NOR gates, a positive voltage can be applied to the drain of the NOR circuit. In practical circuit-level applications, device and process variations must be taken into account, which may necessitate a larger operating voltage. Under such conditions, employing larger load resistors or adding a buffer can serve as an effective measure to secure sufficient output levels. Despite these considerations, the primary goal of this study was to investigate the potential for low-power operation. Accordingly, both the NAND and NOR circuits were designed to function at a supply voltage as low as 0.9 V and −1 V, demonstrating the feasibility of low-power operation.

Reconfigurable silicon transistor operates based on a positive-feedback mechanism. Positive-feedback mechanisms can be classified into two types: potential barrier modulation [13,15] and weak impact ionization [20,21]. Table 1 compares several devices employing these mechanisms. Devices based on weak impact ionization exhibit small subthreshold swings (SS) and abrupt switching characteristics but require high programming voltages. In contrast, previously reported devices utilizing potential barrier modulation achieved steep switching while operating at lower programming voltages. Our proposed device also employs potential barrier modulation and supports both p- and n-channel operation within a single device. This provides advantages in terms of integration density and logic-gate implementation, while achieving a higher on/off current ratio (I_on_/I_off_) compared to previous devices. Furthermore, the proposed device exhibits a significant hysteresis characteristic induced by the positive-feedback operation, which enables simultaneous logic and memory functionalities within the same structure. This feature provides a distinct advantage for logic-in-memory applications.

## 4. Conclusions

In this study, we demonstrated a reconfigurable silicon transistor based on a p-i-n structure, aimed at implementing an LIM architecture to overcome the limitations of the von Neumann structure, and verified its superior characteristics using 2D TCAD simulations. The proposed device could be perfectly reconfigured into p-channel and n-channel modes solely by controlling the polarity of the PG voltage. This reconfigurable operation provided hardware-level flexibility, which reduced the circuit complexity and improved the integration density. Moreover, based on the positive feedback and latch-up phenomena, the device achieved a steep SS of approximately 1 mV/dec and a high ON/OFF current ratio of the order of 10^10^, signifying that a clear switching operation was possible under ultralow power conditions. Building on these characteristics, we successfully implemented a universal logic gate by connecting two identical devices in parallel, demonstrating NAND LIM operation in p-channel mode and NOR LIM operation in n-channel mode within a single-device structure. In conclusion, the proposed device is highly suitable for LIM operations and enables computational and memory functions without requiring data movement. It provides a universal gate that is hardware-reconfigurable and possesses ultralow-power switching characteristics. The findings of this study are expected to contribute to the core device technology for future high-density, low-power next-generation computing systems.

## Figures and Tables

**Table 1:** Evaluation of devices utilizing positive feedback mechanisms.

Ref.	Operating Principle	Minimum SS (mV/dec)	Programming Voltage (|V_DD_|)	I_on_/I_off_	Hysteresis	Year
[12]	Potential barrier modulation	~1	1.3	10^9^	Significant	2022
[13]	Potential barrier modulation	1.36	1	~10^5^	Insignificant	2019
[20]	Weak impact ionization	0.058	1.3	~10^8^	Insignificant	2010
[21]	Weak impact ionization	~0.01	6	~10^4^	Significant	2016
This work	Potential barrier modulation	~0.2	0.9	~10^11^	Significant	2025

## Data Availability

Data are contained within the article.

## Abbreviations

The following abbreviations are used in this manuscript:

BGN	Bandgap narrowing
CG	Control gate
FBFET	Feedback field-effect transistor
LIM	Logic-in-memory
PG	Polarity gate
SS	Subthreshold swing
TCAD	Technology computer-aided design
